# A Novel Fabrication Technique for Liquid-Tight Microchannels by Combination of a Paraffin Polymer and a Photo-Curable Silicone Elastomer

**DOI:** 10.3390/ma9080621

**Published:** 2016-07-27

**Authors:** Katsuo Mogi, Kenshiro Sakata, Yuki Hashimoto, Takatoki Yamamoto

**Affiliations:** Department of Mechanical Engineering, Tokyo Institute of Technology, Tokyo 152-8552, Japan; sakata.k.ac@m.titech.ac.jp (K.S.); hashimoto.y.ai@m.titech.ac.jp (Y.H.)

**Keywords:** microfluidics, microfabrication, paraffin polymer, photo-curable silicone elastomer

## Abstract

The development and growth of microfluidics has been mainly based on various novel fabrication techniques for downsizing and integration of the micro/nano components. Especially, an effective fabrication technique of three-dimensional structures still continues to be strongly required in order to improve device performance, functionality, and device packing density because the conventional lamination-based technique for integrating several two-dimensional components is not enough to satisfy the requirement. Although three-dimensional printers have a high potential for becoming an effective tool to fabricate a three-dimensional microstructure, a leak caused by the roughness of a low-precision structure made by a 3D printer is a critical problem when the microfluidic device is composed of several parts. To build a liquid-tight microchannel on such a low-precision structure, we developed a novel assembly technique in which a paraffin polymer was used as a mold for a microchannel of photo-curable silicone elastomer on a rough surface. The shape and roughness of the molded microchannel was in good agreement with the master pattern. Additionally, the seal performance of the microchannel was demonstrated by an experiment of electrophoresis in the microchannel built on a substrate which has a huge roughness and a joint.

## 1. Introduction

Microfluidic devices have become key tools in many fields, including analytical chemistry, life sciences, and medicine, in conjunction with the ongoing development and increased utilization of labs-on-a-chip and micro total analysis systems (µTAS) [1,2,3,4,5]. This growth has primarily been based on various novel fabrication techniques [6,7,8] for downsizing and integrating all of the micro/nanocomponents necessary for a given function, such as a reaction or analysis. These components include both fluid-control elements (pumps, valves, etc.) and functional elements (heaters, separation structures, and so on). Bioanalytical devices for DNA sequencing and protein analysis were the first major application of microfluidic devices [9,10,11,12], but these devices have been rapidly adopted in other scientific areas, especially in collaborations between medicine and engineering [13]. The current trend is growth in point-of-care, personalized medicine and medical implant devices, and an expanding range of component materials [8,14,15].

In this context, basic studies regarding microprocessing technologies and materials that will enable effective fabrication of three-dimensional (3D) microchannel structures are required to improve the performance, functionality, and packing density of these devices [5,16,17,18,19]. In some cases, two-dimensional microchannels have been patterned on substrates made of glass or polymeric materials, including heat-curable silicone elastomers. These substrates are typically laminated together to obtain the desired three-dimensional structures. However, lamination is insufficient to satisfy the requirements of all three-dimensional structures because the extensibility of such structures is limited to the direction parallel to the substrate surface.

Recently, it has been reported that 3D channel structures can be rapidly fabricated using 3D printers, which have become popular as they represent convenient manufacturing tools. Additionally, the specific functions of microfluidic systems can be easily expanded based on both the lamination of two-dimensional microfabricated substrates and the assembly of 3D parts made of more than one material. Examples include transparent microchannels made of glass to allow for observations, high conductivity surfaces made of metals and acting as electrodes, and flexible structures made of silicone. Despite these advantages, fluid leaks originating at the joints between rough surfaces remain a critical problem associated with microfluidic devices composed of several parts and with imprecise structures.

In the present study, we developed and investigated a novel fabrication technique to address this challenge. A paraffin-based polymer and a photo-curable silicone elastomer were employed to fabricate a liquid-tight microchannel on a rough surface, with the paraffin polymer serving as a mold for the microchannel, which was made of silicone elastomer. The shape transfer capability of the paraffin polymer mold was assessed by evaluating the surface roughness of the finished product. Additionally, the performance of the liquid-tight microchannel between the microchannel substrate made of silicone elastomer and the bottom substrate made of acrylonitrile butadiene styrene resin (ABS) resin was verified with a demonstration of electrophoresis in the microchannel.

## 2. Materials and Methods

**Figure 1:**
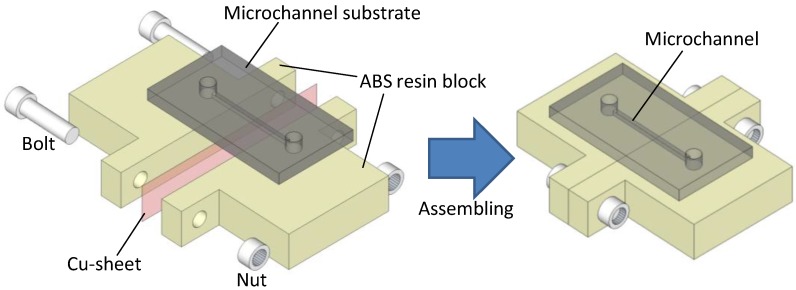
Schematic of the microfluidic device used during the leak test. The silicone substrate had a microchannel with a height of 100 µm, a width of 400 µm and a length of 3 mm. The bottom surface of the channel was composed of two ABS resin blocks and a copper sheet sandwiched between the blocks using two nut and bolt pairs.

To verify the proposed fabrication technique, we demonstrated electrophoresis in a microfluidic device made by the technique shown in Figure 1. This device incorporated a straight microchannel with a height of 90 µm, a width of 500 µm and a length of 3 mm. The lower surface of the channel was composed of two ABS resin blocks, each 30 × 30 × 5 mm^3^ and fabricated using a 3D printer (Mojo 3D printer, Stratasys Inc., Eden Prairie, MN, USA). A 50-µm-thick copper sheet was used as the electrode during electrophoresis, sandwiched tightly between the blocks using two nuts and two bolts.

**Figure 2:**
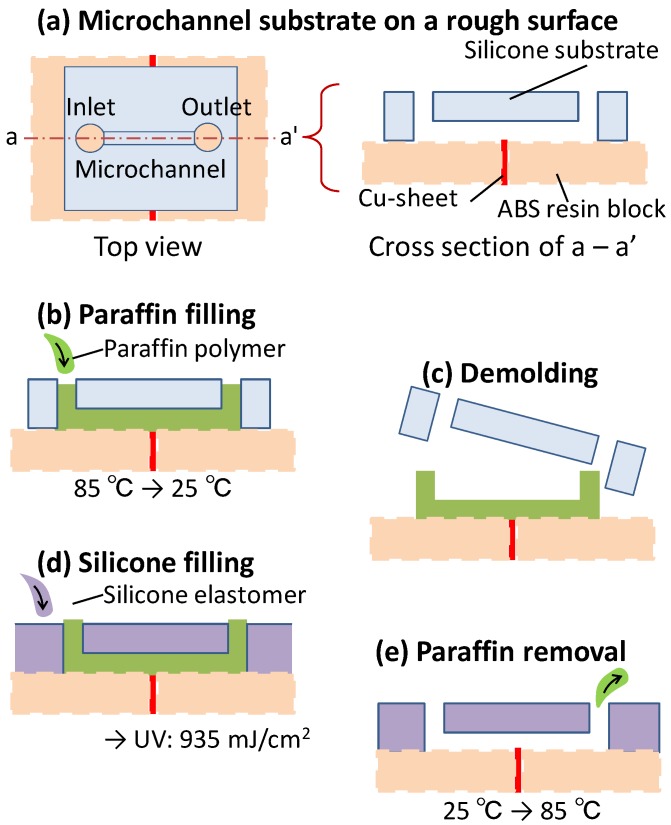
Schematic of the liquid-tight microchannel fabrication process. (**a**) The microchannel substrate made of silicone elastomer on a structure composed of a copper sheet and ABS resin blocks; (**b**) The injection of liquid paraffin polymer into the microchannel at 85 °C; (**c**) Demolding of the microchannel substrate after cooling to room temperature (25 °C), at which point the paraffin polymer is a solid; (**d**) Filling with the photo-curable silicone elastomer following by exposure to UV radiation at 935 mJ/cm^2^; (**e**) Removing the liquid paraffin polymer (molten after heating to 85 °C) after curing the elastomer.

Figure 2 summarizes the proposed technique for the creation of a liquid-tight microchannel. The initial microchannel structure on the silicone substrate is made using a conventional lithography technique [20]. Although the structure on the substrate is typically employed directly as the microchannel, it is important to consider the possibility of liquid leaks in the case that the bottom substrate contains joints between various parts or exhibits significant roughness (Figure 2a).

In the first step of the proposed technique, a paraffin polymer (Hayashi Pure Chemical Ind. Co., Ltd., Osaka, Japan) is heated to 85 °C and injected into the microchannel mold, which is sealed at its base by the bottom substrate, as shown in Figure 2b. A key point here is that the microchannel mold must be held down tightly on the substrate using a rubber band prior to injecting the paraffin. After cooling the paraffin polymer to room temperature, the microchannel substrate is peeled away from the substrate as in Figure 2c. The microchannel structure is now transferred to the paraffin polymer on the substrate used to help form the microchannel, even if the substrate includes joints or has significant roughness. As shown in Figure 2d, the photo-curable silicone elastomer (X-34-4184, Shin-Etsu Silicone, Tokyo, Japan) is then cast into the mold to seal any possible spaces that could result in leaks along the bottom substrate. This elastomer is subsequently cured without the application of high temperature, by UV light irradiation at an energy of 0.94 J/cm^2^, using a low-pressure mercury lamp (Multilight: ML-251, Ushio Inc., Tokyo, Japan) equipped with a 365 nm wavelength bandpass filter [21]. This low temperature fabrication process maintains the shape of the microchannel structure formed by the low melting point paraffin polymer. Additionally, gaps between the elastomer and the bottom surface caused by distortion of the elastomer are avoided owing to the unique low-shrinkage characteristics of the elastomer. Following 30 min of light exposure, the paraffin polymer in the microchannel is removed by heating the apparatus to 85 °C (Figure 2e).

## 3. Experimental Section

To verify the applicability of the proposed technique for microfabrication, the shape transfer capability of the fabricated microchannel was evaluated as an indicator of the accuracy of this process. This was accomplished by assessing the shape and roughness of the paraffin polymer mold and the channel using laser microscopy (VK-X210, Keyence, Osaka, Japan).

**Figure 3:**
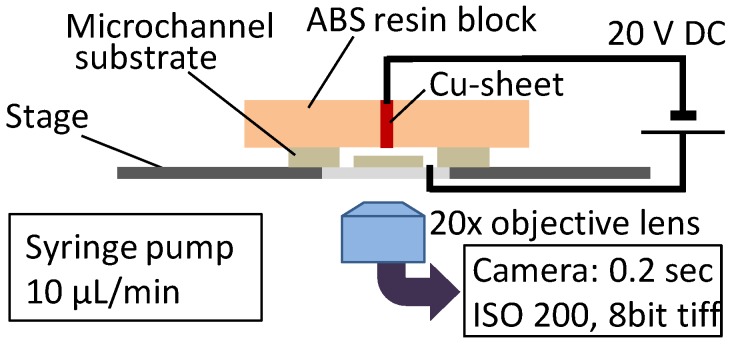
Schematic of the imaging system used during electrophoresis. The device was placed upside down on the microscope stage and the negative terminal of an electronic power supply was connected to the copper sheet, while the positive terminal was connected to a wire in the outlet of the channel. The resulting fluorescence was obtained as eight-bit tiff images.

Additionally, to estimate the sealing performance of the fabricated microchannel, we performed the electrophoresis of a fluorescent molecule (fluorescein, F1300, Molecular Probes, Inc., Eugene, OR, USA) dissolved in distilled water at 10 µg/mL. The fluorescent emission of the fluorescein, as assessed using a fluorescent microscopy system (Microscope: IX 73, Olympus, Tokyo, Japan; Camera: DP72, Olympus), was used to visualize leakage of the solution from the microchannel. During these leak tests, the fluorescein solution was injected into the microchannel at a rate of 10 mL/min via a syringe pump (Kd Scientific, Holliston, MA, USA) and a voltage of 20 V was applied to initiate electrophoresis. The negative terminal of an electronic power supply (P4K-80M, Matsusada, Shiga, Japan) was connected to the copper sheet of the device, while the positive terminal was connected to a wire placed in the outlet of the channel, as shown in Figure 3.

## 4. Results and Discussion

**Figure 4:**
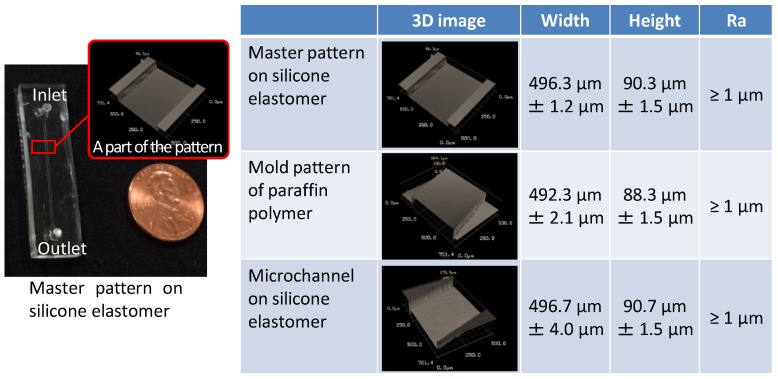
Dimensional and roughness data for a microchannel pattern. The leftmost image is a photograph of the master pattern on the silicon elastomer. The right table shows 3D images at the various stages, the height and width of each structure, and the arithmetic average of the roughness (*Ra*) over a 300 × 300 µm^2^ area.

Figure 4 shows microscopy images of the channel structure fabricated by the proposed technique, applying a master pattern with a width of 496.3 µm and a height of 90.3 µm. Although the dimensions of the molded paraffin presented in this figure are 0.8%–2.2% smaller than the master pattern, the completed microchannel on the silicone elastomer was in good agreement with the master pattern, and the difference is 0.1%–0.4%. It is therefore likely that the apparent difference in the paraffin pattern dimensions was simply a measurement error.

The arithmetic average of the roughness (*Ra*) over a 300 × 300 µm^2^ region of each of these patterns was less than 1 µm, which was the minimum value of roughness resolution in the measurement system employed. Although we consider that additional evaluation depending on the intended application will be necessary in future, the roughness resolution obtained in the present study was sufficient for the purposes of our demonstration. These results indicate that the master pattern was successfully transferred to the paraffin polymer and subsequently to the silicone elastomer, the dimensions of which were in good agreement with those of the master pattern.

**Figure 5:**
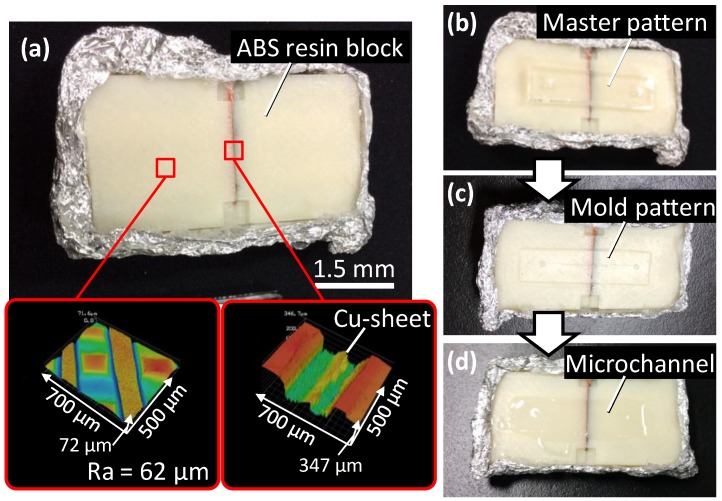
Photographic images of the experimental substrates. (**a**) The bottom substrate composed of ABS resin block. Red squares show magnified images of the surface with a *Ra* of 62 µm and the joint between the copper sheet and ABS resin blocks; (**b**) The master pattern on the bottom substrate; (**c**) The transferred mold pattern on the paraffin polymer; (**d**) The completed microfluidic device composed of a microchannel in a silicone elastomer with an electrode.

Figure 5 presents images of the actual substrates during this process. Figure 5a shows the bottom substrate, composed of a copper sheet and an ABS resin block, the surface of which had a net-like morphology with a *Ra* of 62 µm over a 1.4 × 1.1 mm^2^ area. The gap at the joint between the copper sheet and the resin block was 175 µm wide and 219 µm deep. The master pattern on the bottom substrate was transferred to the silicone elastomer through the molded pattern of the paraffin polymer, as shown in Figure 5b–d.

**Figure 6:**
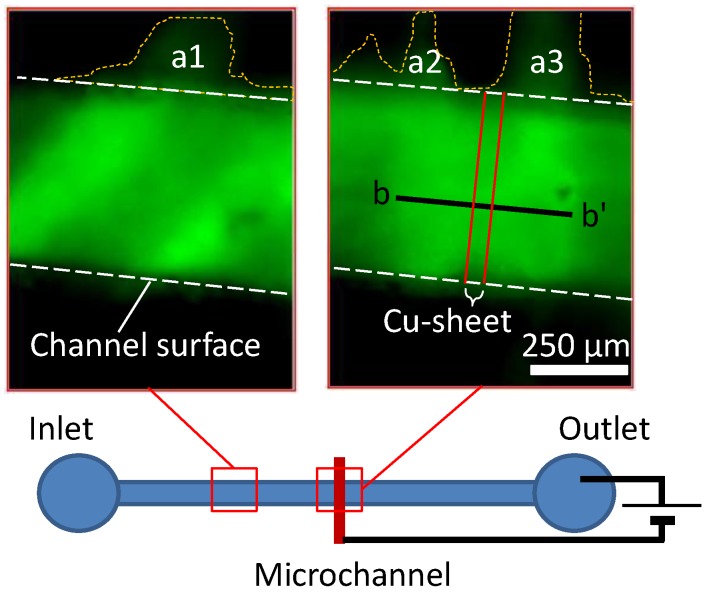
Fluorescent images of a fluorescein solution in the microchannel. The boundary of the channel surface is indicated by the white dotted lines. The relatively small amounts of the fluorescein at a1, a2 and a3 indicate negligible dead end space.

Electrophoresis was demonstrated utilizing the microchannel of the fabricated device. In the case of a microchannel substrate made using a conventional technique, it would be impossible to confirm any structures, including the boundaries of the microchannel, because the significant roughness of the bottom substrate would have resulted in leaks of the injected solution. In the present device, however, the fluorescent image of the fluorescein in the microchannel clearly indicated the boundary of the channel surface in the microchannel made by this new technique, as shown in Figure 6. In these images, the variable intensity in the microchannel indicates a difference in depth resulting from the roughness of the bottom substrate. Although a small amount of fluorescein was confirmed to have leaked to the exterior of the channel (as indicated at the sites labeled a1, a2 and a3 in Figure 6), the fluorescent intensity and apparent flow of the fluorescein in the microchannel demonstrate that there was negligible dead end space.

**Figure 7:**
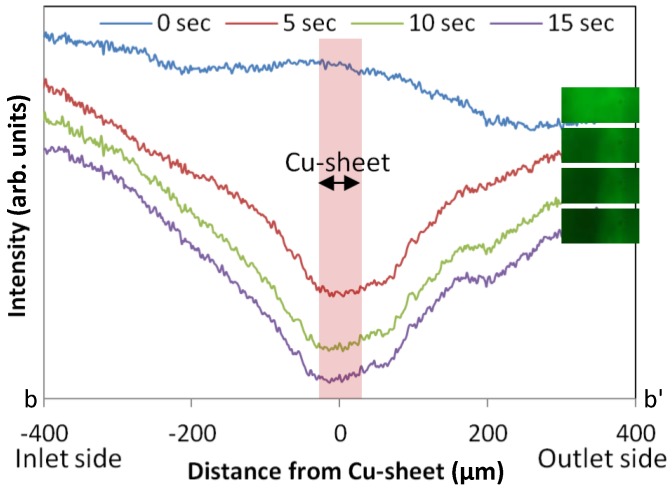
Result of the electrophoresis of fluorescein in the microchannel over 15 s. The *y*-axis shows signal intensity while the *x*-axis indicates the distance from the copper sheet after applying voltage.

Figure 7 presents the result of the electrophoresis of fluorescein in the microchannel. The change in the signal intensity, which was obtained by subtracting a background, was measured for 15 s after applying a voltage to the line between b and b’ indicated in Figure 6. Over the first 5 s, the intensity rapidly decreased in the vicinity of the copper sheet due to electrophoresis. It is evident from this result that the proposed technique has the potential to fabricate unique structures using several different materials for various applications.

## 5. Conclusions

As a means of improving the 3D construction of microfluidic devices, we developed a novel technique for fabricating liquid-tight microchannels between two different device components. Using this method, a microchannel was formed using a photo-curable silicone polymer and was tightly sealed against a bottom substrate which had both joined parts and significant roughness. From measurements of the microchannel dimensions and the roughness of the transferred structure, it was evident that the master pattern was successfully transferred first to the paraffin polymer with a 0.8%–2.2% difference and then to the silicone elastomer with a 0.1%–0.4% difference, which was in good agreement with the dimensions of the master pattern. A liquid-tight microchannel of photo-curable silicone was successfully fabricated on this very rough substrate (*Ra* = 62 µm). As the operation example of a microfluidic device which has a three-dimensional structure composed of several parts such as an electrode of copper, a bottom substrate of ABS resin and a microchannel of silicone, an experiment of electrophoresis in the microchannel was demonstrated.

We believe that the microfabrication technique presented in this paper will have a wide range of applications regarding the construction of unique structures composed of several different materials for various end uses.

## References

[1] Mogi K., Fujii T. (2010). A microfluidic device for stepwise size-based capturing of suspended particles. J. Micromech. Microeng..

[2] Arata H.F., Kumemura M., Sakaki N., Fujita H. (2008). Towards single biomolecule handling and characterization by mems. Anal. Bioanal. Chem..

[3] Craighead H. (2006). Future lab-on-a-chip technologies for interrogating individual molecules. Nature.

[4] Mohammed M.I., Desmulliez M.P. (2011). Lab-on-a-chip based immunosensor principles and technologies for the detection of cardiac biomarkers: A review. Lab Chip.

[5] Chin L.K., Yu J.Q., Fu Y., Yu T., Liu A.Q., Luo K.Q. (2011). Production of reactive oxygen species in endothelial cells under different pulsatile shear stresses and glucose concentrations. Lab Chip.

[6] Miccio L., Memmolo P., Grilli S., Ferraro P. (2012). All-Optical Microfluidic Chips for Reconfigurable Dielectrophoretic Trapping through Slm Light Induced Patterning. Lab Chip.

[7] Mogi K., Fujii T. (2013). A Novel Assembly Technique with Semi-Automatic Alignment for PDMS Substrates. Lab Chip.

[8] Mogi K., Sugii Y., Yamamoto T., Fujii T. (2014). Rapid fabrication technique of nano/microfluidic device with high mechanical stability utilizing two-step soft lithography. Sens. Actuators B: Chem..

[9] Lagally E.T., Medintz I., Mathies R.A. (2001). Single-molecule DNA amplification and analysis in an integrated microfluidic device. Anal. Chem..

[10] Khandurina J., McKnight T.E., Jacobson S.C., Waters L.C., Foote R.S., Ramsey J.M. (2000). Integrated system for rapid pcr-based DNA analysis in microfluidic devices. Anal. Chem..

[11] Zheng B., Roach L.S., Ismagilov R.F. (2003). Screening of protein crystallization conditions on a microfluidic chip using nanoliter-size droplets. J. Am. Chem. Soc..

[12] Urushidani M., Sugii Y., Mogi K., Hishida K. Investigation of Near Surface Flow Field and Glycocalyx Dimension of Endothelial Cells by Confocal Micro-PIV and Super-Resolution Microscopy. Proceedings of the 17th International Symposium on Applications of Laser Techniques to Fluid Mechanics.

[13] Myers F.B., Lee L.P. (2008). Innovations in optical microfluidic technologies for point-of-care diagnostics. Lab Chip.

[14] Carlborg C.F., Haraldsson T., Oberg K., Malkoch M., van der Wijngaart W. (2011). Beyond pdms: Off-stoichiometry thiol-ene (oste) based soft lithography for rapid prototyping of microfluidic devices. Lab Chip.

[15] Daridon A., Fascio V., Lichtenberg J., Wutrich R., Langen H., Verpoorte E., de Rooij N.F. (2001). Multi-layer microfluidic glass chips for microanalytical applications. Fresenius J. Anal. Chem..

[16] Yu M., Wang Q., Patterson J.E., Woolley A.T. (2011). Multilayer polymer microchip capillary array electrophoresis devices with integrated on-chip labeling for high-throughput protein analysis. Anal. Chem..

[17] Thorsen T., Maerkl S.J., Quake S.R. (2002). Microfluidic large-scale integration. Science.

[18] Gerasopoulos K., McCarthy M., Royston E., Culver J.N., Ghodssi R. (2008). Nanostructured nickel electrodes using the tobacco mosaic virus for microbattery applications. J. Micromech. Microeng..

[19] Lee K.S., Boccazzi P., Sinskey A.J., Ram R.J. (2011). Microfluidic chemostat and turbidostat with flow rate, oxygen, and temperature control for dynamic continuous culture. Lab Chip.

[20] Whitesides G.M., Ostuni E., Takayama S., Jiang X., Ingber D.E. (2001). Soft lithography in biology and biochemistry. Annu. Rev. Biomed. Eng..

[21] Mogi K., Hashimoto Y., Tsukahara T., Terano M., Yoshino M., Yamamoto T. (2015). Nanometer-level high-accuracy molding using a photo-curable silicone elastomer by suppressing thermal shrinkage. RSC Adv..

